# Analysis of Risk Factors Associated With Poor Outcome in Posterior Reversible Encephalopathy Syndrome After Treatment in Children: Systematic Review and Meta-Analysis

**DOI:** 10.3389/fneur.2020.00938

**Published:** 2020-08-26

**Authors:** Marady Hun, Jidong Tian, Min Xie, Zhou She, Amin Sheikh Abdirahman, Phanna Han, Wuqing Wan, Chuan Wen

**Affiliations:** ^1^Division of Hematology and Tumor, Children's Medical Center, The Second Xiangya Hospital, Central South University, Changsha, China; ^2^Department of Ophthalmology, The Second Xiangya Hospital, Central South University, Changsha, China

**Keywords:** children, posterior reversible encephalopathy syndrome (PRES), chemotherapy, hematopoietic stem cell transplantation (HSCT), oncology, risk factors, outcome

## Abstract

**Objective:** Chemotherapy and hematopoietic stem cell transplantation (HSCT) play important roles in clinical etiology, symptoms, signs, imaging findings, and biochemical parameters for inducing posterior reversible encephalopathy syndrome (PRES) in pediatric oncologic diseases. We aimed to evaluate various risk factors of pediatric oncologic diseases after conducting chemotherapy and HSCT to induce PRES for predicting the clinical prognosis frequency.

**Methods:** The literature was performed on PubMed, Web of Science, and Embase databases to recognize the qualified studies. The odds ratios (ORs) of related risk factors and their corresponding 95% confidence intervals (CIs) were used to compute the pooled assessments of the outcomes.

**Results:** Six studies were included in the meta-analysis, involving 828 records. The risk of female children has a significantly higher incidence than male children in oncologic age groups of PRES. Children over the age of 10 years old in oncologic age groups develop a significantly increased risk of PRES. Acute graft-versus-host disease (GVHD) has a significant promotion effect on the occurrence of PRES. Hypertension can promote the occurrence of PRES in children. The risk of PRES in immunodeficient children increases significantly. Children with sickle cell disease (SCD) have a significantly increased risk of PRES. The risk of PRES in children with T-cell leukemia rises considerably. The central nervous system (CNS) leukemia/involvement has a significant role in promoting the occurrence of PRES in children. The pooled OR for the factors male, ≥ 10 years old of age, acute GVHD, hypertension, immunodeficiency, SCD, T-cell leukemia, CNS leukemia/involvement was 0.66 (95% CI: 0.58, 0.76; *P* < 0.00001), 2.06 (95% CI: 1.23, 3.43; *P* < 0.006), 1.32 (95% CI: 1.14, 1.53; *P* < 0.0003), 8.84 (95% CI: 7.57, 10.32; *P* < 0.00001), 2.72 (95% CI: 1.81, 4.08; *P* < 0.00001), 2.87 (95% CI: 2.15, 3.83; *P* < 0.00001), 2.84 (95% CI: 1.65, 4.88; *P* < 0.0002), and 3.13 (95% CI: 1.43, 6.84; *P* < 0.004), respectively.

**Conclusions:** The result of this meta-analysis suggests that female children, age over 10 years old, acute GVHD, hypertension, immunodeficiency, SCD, T-cell leukemia, and CNS leukemia/involvement are likely to have the poor outcome in pediatric oncologic/hematologic diseases in PRES.

## Introduction

Posterior reversible encephalopathy syndrome (PRES) is a clinical radiographic syndrome that has been recognized for more than two decades by Dr. Hinchey and his other colleagues by using 15 reports with the different primary diagnoses in adults since 1996 (1). PRES is typically characterized by acute neurological symptoms, such as seizures, mental impairment, visual disturbance, and headache (1, 2), classical vasogenic edema and bilateral, subcortical of focal neurologic lesion sites (e.g., parieto-occipital, frontal, temporal, cerebellar, basal ganglia, brainstem) (3–7).

There are multiple risk factors that contribute to developing PRES, including hypertension, solid organ transplantation, bone marrow transplant, renal disorder, malignancies, systematic connective tissue disorder, blood transfusion, sepsis, hypomagnesemia, severe sepsis, multiple organ dysfunctional syndromes, sickle cell anemia (8), pregnancy (4, 9–12), poisoning (13), etc. PRES should be recognized early to avoid developing complicated conditions or worse, severe neurological sequelae and death (5, 14, 15). Recently a high risk of morbidity and mortality rate of (3.2%) in PRES was reported in a total of 825 children and adolescents in 2019 (8). Mortality was slightly higher at 2.6% in a total of 112 children who underwent hematopoietic stem cell transplantation recipients in 2017 (16). Moreover, a comparative study in PRES between children and adults found that affected children were more likely to suffer from multi-organ failure than affected adults (17). In contrast, a previous study reported that 17.1% of PRES patients died, with the deaths more linked to the risk factors associated with PRES than PRES itself (15).

Despite several recent studies in which the impact of various risk factors on PRES were investigated, the consensus and position statement on these factors on the prognosis of PRES in childhood cancer have still been based on expert opinion and not empirical evidence (2, 8, 16, 18–26). Therefore, we believe that our studies are warranted by a more focused review of the literature. We have used meta-analysis to perform the systematic review for evaluating the association between risk factors and clinical outcomes in pediatric oncologic diseases after conducting chemotherapy and HSCT with PRES.

## Methods

### Search Strategy

In March 2020, the PubMed, Web of Science, and Embase databases were searched.

In PubMed the following searching strategy was used (prognosis OR outcome OR survival OR mortality OR risk factors) AND (posterior leukoencephalopathy syndrome OR posterior reversible encephalopathy syndrome OR reversible posterior leukoencephalopathy syndrome OR PRES OR RPLS) AND (child OR children OR childhood).

In Web of Science the following search strategy was used: #1.Topic (posterior leukoencephalopathy syndrome) OR (posterior reversible encephalopathy syndrome) OR (reversible posterior leukoencephalopathy) OR (PRES) OR (RPLS); #2.Topic (child) OR (children) OR (childhood); #3.Topic (prognosis) OR (outcome) OR (survival) OR (mortality) OR (risk factors).

In Embase the following searching strategy was used: (“posterior leukoencephalopathy syndrome”:ab,ti OR “posterior reversible encephalopathy syndrome”:ab,ti OR “reversible posterior leukoencephalopathy syndrome”:ab,ti OR pres:ab,ti OR rpls:ab,ti) AND (child:ab,ti OR children:ab,ti OR childhood:ab,ti) AND (prognosis:ab,ti OR outcome:ab,ti OR survival:ab,ti OR mortality:ab,ti OR 'risk factor':ab,ti).

### Selection Strategy

Inclusion criteria were used to screen related literature.

1. All included studies were case-control or retrospective studies with sample sizes of at least 20 or more cases of children (participants <20 years of age) with PRES.
2. The included studies must report the odds ratio (OR) of related risk factors and its 95% confidence interval (CI). In addition, the researchers must provide sufficient data to calculate the OR and its 95% CI of child-related risk factors.
3. All the included studies had to estimate the association between the related risk factors, including clinical etiology, symptoms, imaging findings, laboratory parameters, and clinical outcome in children with PRES.
4. All the studies published in the same research team had to use different patient data, especially in oncologic pediatric patients.
5. The studies included had to be original, and reviews, case reports, commentaries, laboratories, abstracts, meta-analyses, and editorials were excluded.
6. Duplicated publication of the literature, incomplete data, and unclear outcome effects, incorrect statistical methods, lack of provision of OR, 95% CIs, diagnostic criteria for PRES, and data on non-children PRES were excluded.
7. The included researches had to be reported in the English language.

### Literature Screening and Data Extraction

Literature screening and data extraction were incorporated in the inclusion and exclusion criteria by two researchers, who read the titles and abstracts of the literature for initial screening. If there are different opinions, an agreement was reached through discussion. A self-designed data extraction table was used to extract the data from the included literature, such as the last name of the first author, the year of publication, the country of research, the sample size from the necessary information of the literature, age-related factors (mean, age group, and median), sex (male), primary diagnoses, etiologies, symptoms/signs, biochemical parameters, mortality rate, and Newcastle-Ottawa Scale (NOS) scores (see Tables 1, 2).

**Table 1:** Quality scores of included studies using newcastle-ottawa scale.

**Author**	**Selection**				**Comparability**	**Outcome**			**NOS**	**Outcome indicator**
	**Representativeness of the exposed**	**Selection of the non-exposed cohort**	**Ascertainment of expose**	**Demonstration that outcome of interest was not present at start of study**	**Comparability of cohort on the basis of the design or analysis**	**Assessment of outcome**	**Was follow-up long enough for outcomes**	**Adequacy of follow-up of cohorts**	**Overall score**	
Thavamni (2019) (8)	☆	☆	☆	☆	☆☆	☆	☆	☆	9 stars	1, 4, 5, 6
Anastasopoulou (2019) (2)	☆	☆	☆	☆	☆☆	☆	☆	☆	9 stars	1, 7, 8
Li (2019) (25)	-	☆	☆	☆	☆☆	☆	☆	☆	8 stars	1, 2, 3,4
Banerjee (2018) (20)	-	☆	☆	☆	☆☆	☆	☆	☆	8 stars	1, 2, 4, 7, 8
Gaziev (2017) (18)	-	-	☆	☆	☆☆	☆	☆	☆	7 stars	1, 3, 4, 6
Zama (2014) (27)	-	☆	☆	☆	☆☆	☆	☆	☆	8 stars	5

*NOS≥ 7 high quality; 1, sex (male); 2, age group ≥ 10 years old (or 10.0-20.0 y); 3, acute GVHD; 4, hypertension; 5, immunodeficiency; 6, sickle cell disease; 7, T-cell leukemia; 8, central nervous system leukemia/involvement. Note: The stars are proved by all NOS scale's questions above. The full score is 9 stars; 1–3 stars are low-quality literature, 4–6 stars are medium-quality literature, and 7–9 stars are high-quality literature*.

**Table 2:** Description of the literature included in this study.

**No**	**Study**	**Sample size**	**Age-related (year)**	**Sex (male)**	**Primary diagnoses**	**Etiologies**	**Symptoms/signs**	**Biochemical parameters**	**Morality rate**	**NOS scores**
1	Thavamani (2019) USA (8)	825/2,295,395	Age group: 1.0–20.0y; Mean: 12.54 ± 0.19y	298/825 (36%)	General pediatric population	Solid organ transplantation status 29 (3.5%); bone marrow transplantation 39 (4.7%); renal disorder 400(48.5%); immunodeficiency 28 (3.4%); malignancies 15 (1.8%); systemic connective tissue disorder 66 (8.0%); blood transfusion 85 (10.3%); sickle cell disease 50 (6.1%); anemia 94 (11.4%)	Hypertension 270 (32.7%); sepsis 48 (5.8%); sepsis/MODS 48 (5.8%)	Hypomagnesemia 73 (8.8%)	26/825 (3.2%)	9 stars
2	Anastasopoulou (2019) Sweden (2)	52/1,326	Age group: 10.0–18.0y; 16/52 (30.8%); Median: 8.5y, (1.8–14.8y)	25/52 (48%)	Acute lymphoblastic leukemia; T-ALL, 15/52 (28.8%)	Chemotherapy (NOPHO- ALL 2008 protocol); relapse 2/52 (3.8%); induction therapy prednisolone 34/52 (65.4%); dexamethasone 18/52 (34.6%)	Seizures 43/52 (82.7%); encephalopathy 33/51 (64.7%); visual change 17/51 (33.3%); pyramidal weakness 14/52 (26.9%); headache 15/51 (29.4%); dysphasia 10/51 (19.6%); nausea 10/50 (20.0%); sensory disturbance 7/51 (13.7%); dyspraxia 3/50 (6.0%); psychosis 1/51 (1.9%); fever 11/50 (22.0%); hypertension 41/52 (78.8%); constipation 27/52 (51.9%); abdominal pain 28/52 (53.8%); pancreatitis 4/36 (11.1%); ileus 1/36 (2.8%); infection 22/49 (44.9%)	Hyponatremia 31/44 (70.5%); Hypocalcemia 18/43 (41.9%); abnormal magnesium (↑or↓) 11/43 (25.6%); Abnormal glucose (↑or ↓) 15/42 (35.7%); Acidosis 12/42 (28.6%) Abnormal findings in liquor 5/22 (22.7%)	2/52 (3.8%)	9 stars
3	Li (2019) China (25)	11/84	Median: 4.0 y, (2.0–12.0 y)	5/11 (45.4%)	Thalassemia	Hematopoietic cell transplantation Cyclosporine*CSA (*n* = 8); Tacrolimus*TAC (*n* = 3); calcineurin inhibitors (13.1%)	Hypertension 77 (91.7%); severe hypertension 43 (55.8%); chronic GVHD 36 (42.9%); acute GVHD 11/11 (100.0%); convulsion (*n* = 13); mental status change (*n* = 15); headache (*n* = 13)	Serum ferritin (≥1,500 ng/ml) 9/11 (81.8%)	2/84 (2.4%)	8 stars
4	Banerjee (2018) Sweden (20)	29/643	Age group: 10.0–18.0 y; 9/29 (31.0%)	17/29 (58.6%)	Acute lymphoblastic leukemia; T-ALL, (17.2%)	Chemotherapy (NOPHO-ALL 92/2000 protocols) Induction 28/29 (96.6%); relapse 13/29 (44.8%)	Seizures 21/29 (72.4%); hypertension 23/29 (79.3%); abdominal pain 21/29 (72.4%); visual disturbance 13/29 (44.8%); headache 5/29 (17.9%)	Hyponatremia 23/26 (88.4%); hypomagnesemia 2/13 (15.4%)	NA	8 stars
5	Gaziev (2017) Italy (18)	31/281	Age group: 10.0–17.8y; 14/31 (45.2%); Median thalassemia: 8.0y, (1.4–17.8y); SCD 10.0 y, (2.0–17.0 y)	23/31 (74.2%)	Thalassemia (18/222); Sickle cell disease (13/59)	Hematopoietic cell transplantation CSA (*n* = 27); TAC (*n* = 4); calcineurin inhibitor (11%)	Hypertension 31 (100%); impaired alertness 20 (65%); seizures 30 (98%); unconsciousness 10 (32%); headache 28 (90%); blurred vision 8 (26%); blindness 4 (13%); nausea/vomiting 18 (58%); upper hands tremor 25 (81%); acute GVHD 16/31 (51.6%)	Serum ferritin (≥1,750 ng/ml) 11/31 (35.5%)	7/31 (22.6%)	7 stars
6	Zama (2014) Italy (27)	26/287	Age group ≥2 y; 23/26 (88.5%)	13/26 (50.0%)	Oncological disease (17/26); non-oncological disease (9/26); hemoglobinopathies (8/26); immunodeficiencies (1/26)	Hematopoietic cell transplantation calcineurin inhibitor 25/26 (96.1%); T-cell depletion 1/26 (3.9%); relapse 20/26 (77.0%)	Acute GVHD 21/26 (80.7%); chronic GVHD 17/26 (56.4%)	NA	NA	8 stars

*PRES, posterior reversible encephalopathy; T-ALL, T-cell acute lymphoblastic leukemia; NOPHO-ALL, Nordic Society of Pediatric Hematology and Oncology acute lymphoblastic leukemia; NOS, Newcastle-Ottawa scale; GVHD, graft-versus-host disease; NA, not applicable*.

* *CSA; TAC, n patients developed PRES while being treated with CSA or TAC*.

### Statistical Analysis

This article uses Review Manager (Rev Man) version 5.3 (http://www.cochrane.org) and STATA15.1 software to carry out statistical analysis of the included data. The risk factors in this study were calculated by ES (95% Cl) for each logarithm OR(log^OR^) as the effective value index. They were recognized by the estimated variance of the log^OR^. The generic inverse variance process was conducted for weighting, and *P* < 0.05 was considered as a statistically significant difference between the groups or subgroups, and forest plots were drawn for related factors. Heterogeneity groups were measured using the “Chi-square and Cochrane Q test” to calculate the *P*-value. If *I*^2^ = 0%, each study can be considered homogeneous. If *I*^2^ is between 0 and 50%, it indicates that the study was heterogeneous. The homogeneity is better, and a fixed-effect model is used for analysis. Conversely, if *I*^2^ is between 50 and 100%, it indicates that the homogeneity between studies is poor, and there is significant heterogeneity between groups (28). A random-effect model is used for analysis. Factors with higher heterogeneity further explore the source of heterogeneity, and if necessary, perform sensitivity analysis, subgroup analysis, or meta-regression. The Funnel plot, Egger's test (29), and Begg's test are used to indicate publication bias. If the graph is symmetrical, the publication bias is not considered.

### Literature Quality Evaluation

The literature in this study is observational cohort studies and was evaluated using the NOS scores (http://www.ohri.ca/programs/clinical_epidemiology/oxford.asp). The quality of observational studies is based on the NOS scale. The full score is 9 stars; 1–3 stars are low-quality literature, 4–6 stars are medium-quality literature, and 7–9 stars are high-quality literature (30) (see Table 1).

## Results

### Characteristics of Included Studies

**Figure 1:**
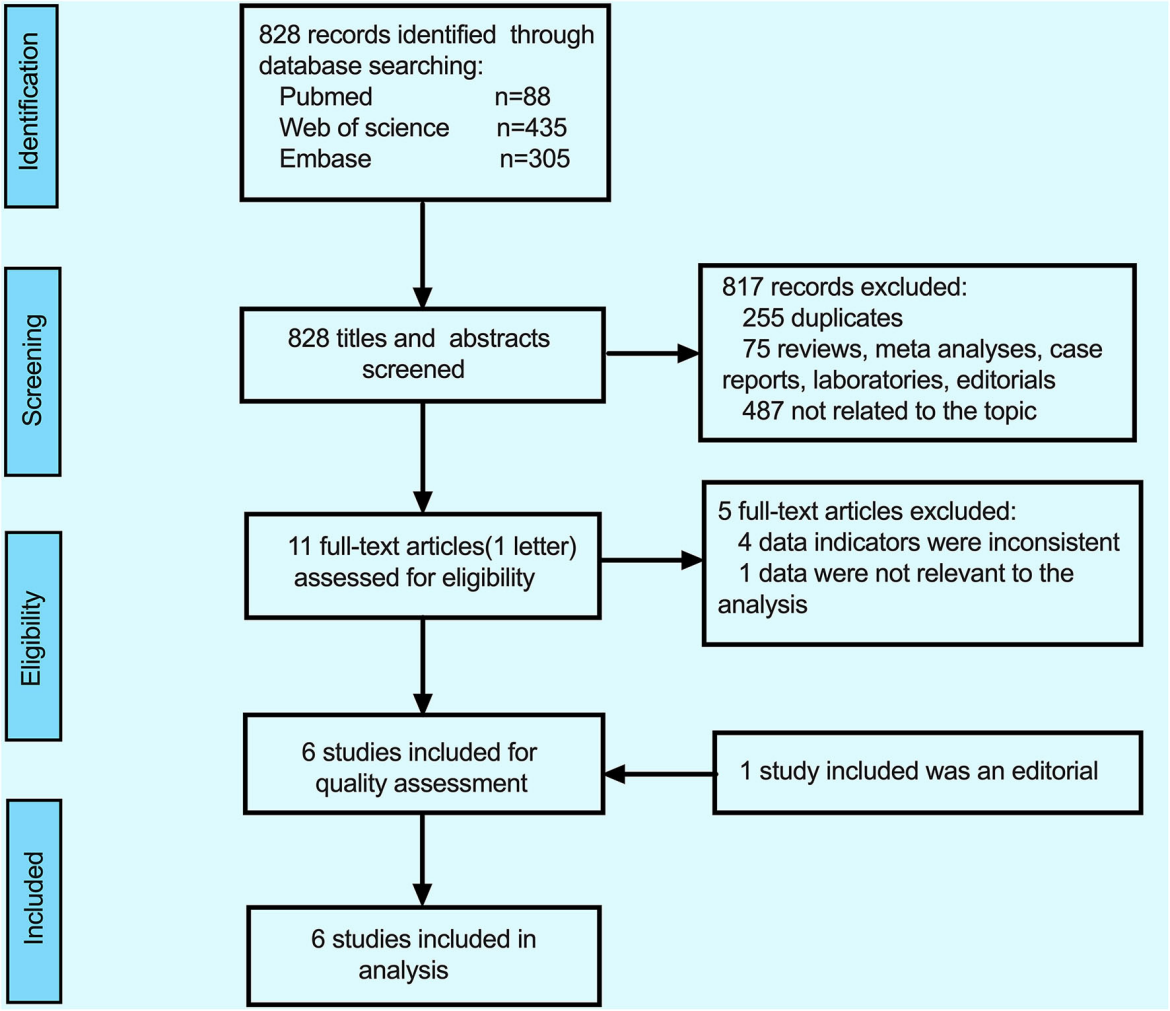
Flowchart of studies selection.

The detailed process of inclusion and exclusion (PRISMA statement) (31) is illustrated by a flow diagram in Figure 1. By searching in the PubMed (*n* = 88), EMBASE (*n* = 305), and Web of Science (*n* = 435) databases updated to March 2020, a total of 828 studies were retrieved on the initial search, 817 studies were excluded, among of 817 studies, 255 duplicates, 75 were reviews+ meta-analyses+ case reports+ laboratories, and 487 were not related to the topic. After the full-text articles of the remaining 11 studies were reviewed, five studies were excluded (the reasons for exclusion are detailed in Figure 1), and eventually, six reports (because of the lack of full-text article, one editorial was included) were included in this meta-analysis.

A total of six studies (2, 8, 18, 20, 25, 27) were selected for meta-analysis, and due to the lack of full-text articles, one of them was included as an editor study (27). All the reviews were observational cohort studies. Of these six studies, two were from Sweden, two were from Italy, one was from the United States of America, and one was from China. Most of the included PRES patients in children (aged <20 years) had primary diagnoses of leukemia, thalassemia, and sickle cell disease that were treated with hematopoietic cell transplantation and chemotherapy following protocols from the different years.

In whole studies, the risk factors of multiple specifications on the clinical outcomes of PRES in children were analyzed. These include sex (male), age group ≥10 years old (or 10.0–20.0 years old), hypertension, sepsis, severe sepsis, multiple organ dysfunction syndrome (MODS), seizures, encephalopathy, headache, constipation, infection, mental disturbance, visual impaired, acute GVHD/chronic GVHD, immunodeficiency, blood transfusion, sickle cell disease, T-cell leukemia, central nervous system leukemia, hemoglobinopathy, and biochemical parameters (hypomagnesemia, hyponatremia, hypocalcemia, serum ferritin level, etc.) (see Table 2). Eight of the above risk factors, namely, sex (male), age group ≥10 years old or (10.0–20.0 years old), acute GVHD, hypertension, immunodeficiency, sickle cell disease, T-cell leukemia, CNS leukemia/involvement were detial in the six included studies, and their ORs and 95% CIs were calculated. Among the six included studies, there were two included studies detailed in sex (female) with OR value and 95% CI, and using inverse numbers to calculate sex (male) with OR value and 95% CI(2, 25). The quality in this study was evaluated with the NOS scale (30) and of these six included studies, two studies got a score of 9, three scored 8, and one scored 7 (see Table 1).

### Meta-Analysis of Included Studies

#### Meta-Analysis of Sex (Male)

**Figure 2:**
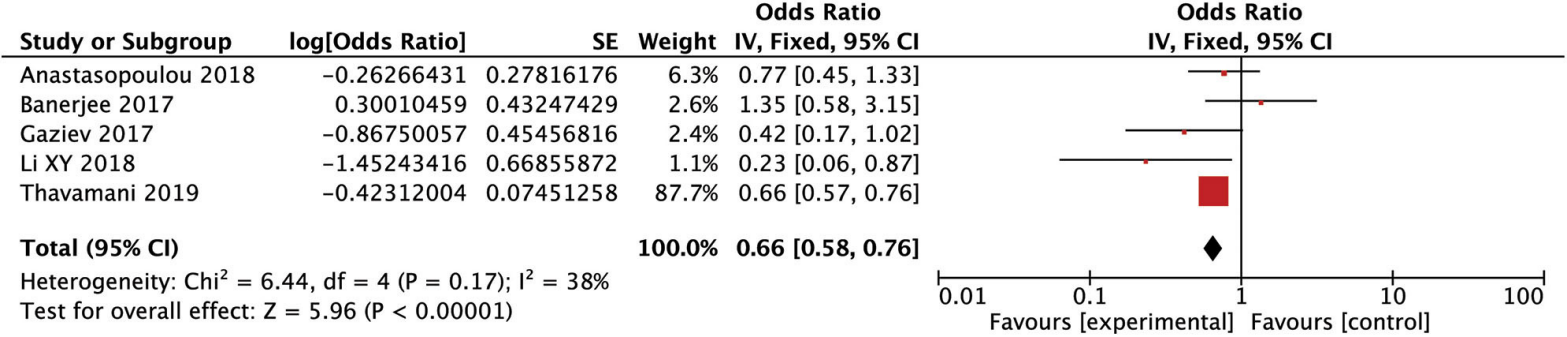
Forest plot for the incidence of sex (male). There is no obvious heterogeneity between the 5 records.

After conducting the heterogeneity test of five records in this study, the results obtained were as follows: Chi^2^ = 6.44, df = 4, *I*^2^ = 38% <50%, and *P* = 0.17 > 0.1 in *Q*-test, suggesting that there is no obvious heterogeneity between the five records using the mixed-effect meta-analysis. The mixed-effect or pooled OR of the five records in this study reached 0.66, 95% CIs (0.58, 0.76), and the results were significant (*Z* = 5.92, *P* = 0.00001 < 0.05), indicating that the risk in female children was significantly higher than in male children (see Figure 2). The sensitivity analysis of the study is shown for sex (male). It can be seen that no literature has a great influence on the results, which indicated that the results of this study are relatedly stable (see Supplementary Figure 1). The five kinds of the literature of this study resulted in the Funnel plot as follows. It can be seen that the Funnel plot is relatively symmetrical, and the Begg's test results (*P* = 0.73 > 0.05) and Egger's test results (*P* = 0.86 > 0.05) based on the Funnel plot illustrate that this study on the five records has no obvious publication bias (see Supplementary Figure 2).

#### Meta-Analysis of Age Group ≥10 Years Old (or 10.0–20.0 Years Old)

**Figure 3:**
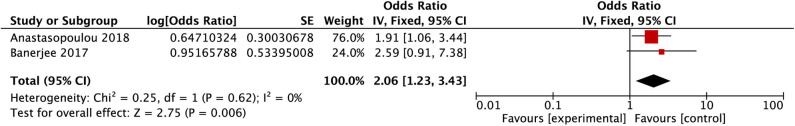
Forest plot for the incidence of age group older than 10 years old. There is no obvious heterogeneity between the 2 records.

After conducting the heterogeneity test of two records in this study, the results obtained were as follows: Chi^2^ = 0.25, df = 1, *I*^2^ = 0 <50%, and *P* = 0.62 > 0.1 in *Q*-test, suggesting that there is no obvious heterogeneity between the two literatures selected using the mixed-effect meta-analysis. The mixed-effect or pooled OR of the two records in this study reached 2.06, 95% CIs (1.23, 3.43), and the results were significant (*Z* = 2.75, *P* = 0.006 < 0.05), indicating that children over the age of 10 years old developed a significantly increased risk of PRES (see Figure 3).

#### Meta-Analysis of Acute GVHD

**Figure 4:**
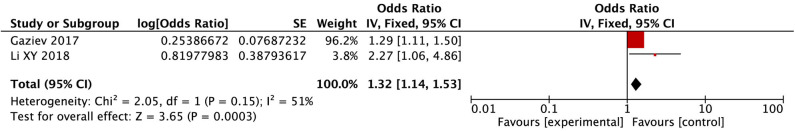
Forest plot for the incidence of acute GVHD. There is no obvious heterogeneity between the 2 records.

After conducting the heterogeneity test of two records in this study, the results obtained were as follows: Chi^2^ = 2.05, df = 1, *I*^2^ = 51% (close to 50%), and *P* = 0.15 > 0.1 in *Q*-test, suggesting that there is no obvious heterogeneity between the literature selected in this study, using mixed-effects meta-analysis. The mixed-effect or pooled OR of the two records in this study reached 1.32, 95% CIs (1.14, 1.53), and the results were significant (*Z* = 3.65, *P* = 0.0003 < 0.05), indicating that acute GVHD has a significant effect on the occurrence of PRES (see Figure 4).

#### Meta-Analysis of Hypertension

**Figure 5:**
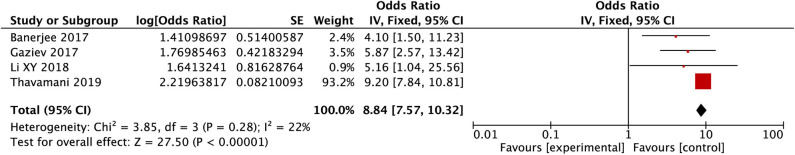
Forest plot for the incidence of hypertension. There is no obvious heterogeneity between the 4 records.

After conducting the heterogeneity test of the four records in this study, the results obtained were as follows: Chi^2^ = 3.85, df = 3, *I*^2^ = 22 < 50%, and *P* = 0.28 > 0.1 in *Q*-test, suggesting that there is no obvious heterogeneity between the four records, using the mixed-effect meta-analysis. The mixed-effect or pooled OR of the four records in this study reached 8.84, 95% CIs (7.57, 10.32), indicating that hypertension could promote the occurrence of PRES in children (see Figure 5). The sensitivity analysis of this study is shown for hypertension. It can be seen that Thavamani's research may have a particular impact on the stability of the results, but it is still within the acceptable range (see Supplementary Figure 3).

#### Meta-Analysis of Immunodeficiency

**Figure 6:**
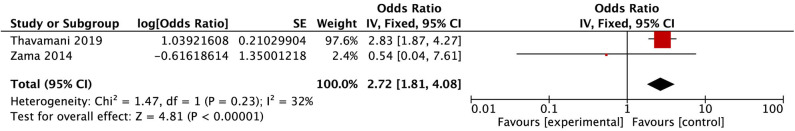
Forest plot for the incidence of immunodeficiency. There is no obvious heterogeneity between the 2 records.

After conducting the heterogeneity test of two records in this study, the results obtained were as follows: Chi^2^ = 1.47, df = 1, *I*^2^ = 32 < 50%, and *P* = 0.23 > 0.1 in *Q*-test, suggesting that there is no obvious heterogeneity between the two literatures selected in this study. The mixed-effect or pooled OR of the two records in this study reached 2.72, 95% CIs (1.81, 4.08), and the results were significant (*Z* = 4.82, *P* = 0.00001 < 0.05), indicating that the risk of PRES in immunodeficiency children was significantly increased (see Figure 6).

#### Meta-Analysis of Sickle Cell Disease

**Figure 7:**
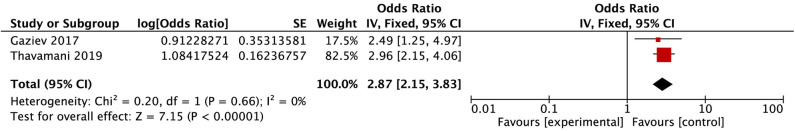
Forest plot for the incidence of SCD. There is no obvious heterogeneity between the 2 records.

After conducting the heterogeneity test of two records in this study, the results obtained were as follows: Chi^2^ = 0.20, df = 1, *I*^2^ = 0 < 50%, and *P* = 0.66 > 0.1 in *Q*-test, suggesting that there is no obvious heterogeneity between the two literatures selected in this study using mixed-effect meta-analysis. The mixed-effect or pooled OR of the two records in this study reached 2.87, 95% CIs (2.15, 3.83), and the results were significant (*Z* = 7.16, *P* = 0.00001 < 0.05), indicating that children with sickle cell disease had a significantly increased risk of PRES (see Figure 7).

#### Meta-Analysis of T-Cell Leukemia

**Figure 8:**
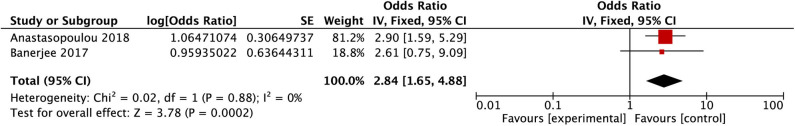
Forest plot for the incidence of T-cell leukemia. There is no obvious heterogeneity between the 2 records.

After conducting the heterogeneity test of two records in this study, the results obtained were as follows: Chi^2^ = 0.02, df = 1, *I*^2^ = 0 < 50%, and *P* = 0.88 > 0.1 in *Q*-test, suggesting that there is no obvious heterogeneity between the two literatures selected in this study using mixed-effect meta-analysis. The mixed-effect or pooled OR of the two records in this study reached 2.84, 95% CIs (1.65, 4.88), and the results were significant (*Z* = 3.79, *P* = 0.0002 < 0.05), indicating that the risk of PRES in children with T-cell leukemia was significantly increased (see Figure 8).

#### Meta-Analysis of Central Nervous System Leukemia/Involvement

**Figure 9:**
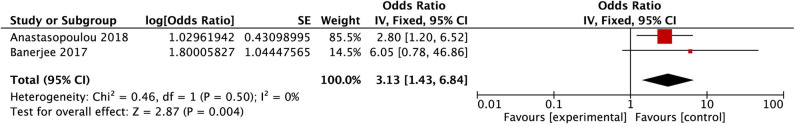
Forest plot for the incidence of CNS leukemia/involvement. There is no obvious heterogeneity between the 2 records.

After conducting the heterogeneity test of two records in this study, the results obtained were as follows: Chi^2^ = 0.46, df = 1, *I*^2^ = 0 < 50%, and *P* = 0.50 > 0.1 in *Q*-test, suggesting that there is no obvious heterogeneity between the two literatures selected in this study using mixed-effect meta-analysis. The mixed-effect or pooled OR of the two records in this study reached 3.13, 95% CIs (1.44, 6.84), and the results were significant (*Z* = 2.87, *P* = 0.004 < 0.05), indicating that CNS leukemia/involvement has a vital role in promoting the occurrence of PRES in children (see Figure 9).

As mentioned above, the risk factors of these six studies, which included sex (male), age group ≥10 years (10.0–20.0 years), hypertension, sepsis, severe sepsis, MODS, seizures, encephalopathy, headache, constipation, infection, mental disturbance, visual impaired, acute GVHD/chronic GVHD, immunodeficiency, blood transfusion, sickle cell disease, T-cell leukemia, central nervous system leukemia, hemoglobinopathy, and biochemical parameters (hypomagnesemia, hyponatremia, hypocalcemia, serum ferritin level, etc.), were also relevant to the risk factors of the adverse outcome of PRES in children, although only eight elements were evaluated among these risk factors, and the others could not be used in this meta-analysis.

## Discussion

This meta-analysis included six studies with a combined study population of 974 patients that were children with PRES. The citation search period of these studies ranged from 1980 to 2020, and these studies were conducted in China, Italy, the USA, Sweden.

Generally, PRES has good prognosis, and clinical symptoms and imaging changes are reversible if diagnosed and treated early (32). However, if the diagnosis or treatment is not made in time, neurological sequelae or even death may occur, especially when combined with intracranial hemorrhage or cerebral infarction (12). Elevated lactic dehydrogenase (LDH) levels and higher blood pressure may predict more substantial and more diffuse PRES lesions. The degree of edema manifestation is likely to reflect the overall severity of the systemic process, and the functions of biochemical parameters and clinical symptoms are significant in determining the prognosis of PRES patients (33–35). The serum LDH, a marker of endothelial dysfunction, shows a statistically significant elevation at the onset of PRES toxicity in cancer patients receiving chemotherapy. The systemic process characterized by endothelial injury/dysfunction as a factor, if not the prime event, in the pathophysiology of PRES, as reported by Fitzgerald et al. (36). Siebert et al. (19) analyzed factors related to the outcome of PRES death and found that changes in mental status, subarachnoid hemorrhage, significantly increased C-creative protein (CRP), and changes in coagulation function were more likely to cause death. Chen et al. (11) used meta-analysis to analyze the evidence of risk factors in adult age groups for PRES, and found that hemorrhage or cytotoxic edema was likely associated with poor outcome in PRES. In contrast, the toxemia of pregnancy (pre-eclampsia/eclampsia) was likely associated with reducing the risk of poor outcome in PRES. Those risk factors mentioned above were definitely found in the adults. Habetz et al. (17) studied 19 pediatric patients and 100 adult patients with PRES to compare the risk factors, and the results suggested that PRES in children was commonly observed in those with multi-organ failure.

Although numerous studies have investigated PRES continuously, the specific risk factors of developing PRES in children remain unclear, since PRES occurs before or during acute neurological symptoms, such as seizures, mental impairment, visual disturbance, headache, vomiting, and other risk factors associated with progressing long-term neurological disorder or death. Of the six studies included (2, 8, 18, 20, 25, 27), we found that data on sex (male), age group ≥10 years (or 10.0–20.0 years), acute GVHD, hypertension, immunodeficiency, sickle cell disease, T-cell leukemia, CNS leukemia/involvement are associated with poor outcomes or prognoses of patients with PRES and is a limitation on children. In this meta-analysis, a significantly higher incidence of risk was found in female children than in male children in the oncologic age groups of PRES. Children over the age of 10 years old in the oncologic age groups, hypertension, acute GVHD, SCD, immunodeficiency, T-cell leukemia, and CNS leukemia/involvement, were factors noted to significantly increase the risk of developing PRES. In our meta-analysis, most of our pediatric PRES's primary diagnoses were collected from oncologic and hematologic diseases that were conducted by HSCT, chemotherapy from the different year protocols.

In a previous study about the demography of PRES patients, a survey by Legriel et al. (4) showed that among 70 patients with PRES, 45 patients (64%) were females. Females tended to have a higher risk than males for developing PRES in adults as reported by Gao et al. (37). However, only a few recent studies of PRES in children mentioned the demography; hence, this assertion remains inconclusive. A study on the general pediatric population with PRES by Thavamani et al. (8) suggested that the mean age at presentation was 12.54 ± 0.19 years, being more common in the adolescent age group, and females tend to be at a higher risk for developing PRES. However, in a 2018 study published by Li et al. (25), in a total of 84 children with the thalassemia after conducting HSCT, 62 patients were male, and of the 11 PRES patients, 5 patients (45.4%) were male. In 2017, a study reported by Gaziev et al. (18) showed that in children with hematological diseases after HSCT, the male-to-female ratio was 23:8, and in pediatric cancers treated with chemotherapy, the male-to-female ratio was 14 (58%):10 (41.7%) among 24 pediatric patients with PRES (7). In contrast, a case-control study composed of acute lymphoblastic leukemia (ALL) pediatric patients that also underwent chemotherapy concluded that male-to-female ratio was 25 (48%):27 (51%), and the age group 10.0–17.0 years, or older children were prone to developing PRES (2). In the same year with the same ALL pediatric patients, among all (*n* = 643) of the patients with PRES (*n* = 29), 12 (41.4%) were females. These inconsistent results of demography may be due to differences in sample size, research population, and primary diagnoses and/or conducting HSCT, chemotherapy, or transplantation in children. Our analysis showed that the risk of female children has a significantly higher incidence than male children in the oncologic age groups of PRES, and children over the age of 10 years develop a significantly increased risk for PRES.

HSCT and chemotherapy can produce toxic reactions to capillary endothelial cells. Damaged vascular endothelial cells release vasoconstrictor substances such as endothelin and prostacyclin. The consequent endothelial activation starts a molecular cascade, cerebral vasospasm contraction, and capillary permeability increased, which finally causes the production of molecules that alter the normal homeostasis of blood-brain-barrier. This alteration consists of a weakening of brain vessel tight junctions, which allows fluid leakage and edema. In this scenario, hypertension would be an epiphenomenon of the underlying mechanism and not the cause, for this reason, it can be present or not in PRES (38, 39). Many scholars believe that in both children and adults, the distribution of hypertension is one of the leading causes of developing PRES (5, 12, 14, 15, 25, 27, 40–42). About 20–65% of PRES patients had hypertension in adults (12), and because the cerebral blood flow (CBF) autoregulation threshold is lower in children than in adults, the mean of blood pressure at the onset of PRES symptoms is lower. In children, the lower limit of CBF autoregulation is 40 mmHg, but in adults, its average is about 50–60 mmHg; as a result, hypertension is more common in pediatric PRES (43). A series of currently published studies, that ranged from 2015 to 2020, investigating PRES-hypertension in pediatric oncology or hematology diseases suggested that ~67–100% of PRES patients had hypertension after undergoing HSCT or chemotherapy (2, 15, 16, 18, 20, 22, 24, 25, 44–46). The result of our analysis suggested that hypertension can promote the occurrence of PRES in children.

Calcineurin inhibitors are mostly used for the prevention and treatment of GVHD after HSCT. Their cytotoxicity can directly damage vascular endothelial cells, thereby changing the permeability of microvessels, and fluid leakage from the blood vessels increases. At the same time, it also causes increase in blood pressure and local cerebral tissue ischemia by constricting cerebral blood vessels, leading to vasogenic brain edema (47). However, GVHD damages vascular endothelial cells by activating macrophages, donor T cells, and releasing proinflammation factors, which further exacerbates vascular endothelial dysfunction, and in turn, leads to PRES (18, 48). Survival in PRES related to GVHD is poor in children because of the high rate of mortality (16, 18, 25, 49). In our analysis, it is suggested that acute GVHD has a significant promotive effect on the occurrence of PRES.

SCD adversely causes life-threatening condition in children, as recently reported (50). Approximately one or more pathophysiologic factors in pediatric SCD patients that induce endothelial dysfunction, including hypoxia, endothelial damage, and chronic hemolysis due to sickling. The harmful effects of calcineurin inhibitors after conducting HSCT, such as proinflammatory and vasoconstriction effects on the endothelium, may exacerbate endothelial dysfunction, making SCD patients more prone to neurological complications, including PRES (51). Early in 2009, a report by Khademian et al. (52) of 80 pediatric patients, PRES and sickle cell disease were found to occur together with a high frequency of 10%. Recent studies on the association between PRES and SCD are few. However, the incidence of 22% in children who underwent HSCT due to a combination of risk factors provided additional evidence that the occurrence of PRES-SCD was significantly higher than PRES-thalassemia in PRES patients. A study investigating the association of the pathophysiology of SCD after HSCT (18) compared another large cohort study in the general pediatric population and found an incidence of 0.1% of PRES-SCD-related hospitalizations for the first time (8). Our analysis suggested that children with sickle cell disease has a significantly increased risk of PRES.

As of date, there seems to be a lack of studies identifying risk factors for pediatric PRES patients related to immunodeficiency. However, there were several studies that found that PRES is related to human immunodeficiency virus (HIV) (53–55). A low incidence of pediatric PRES patients (1.5%) after HSCT with immunodeficiency was found by Zama et al. (27) in 2014. While in a total of 21 PRES patients, accounting for seven patients (33%) had immunodeficiency in children after HSCT reported by Dandoy et al. (56) in 2015. Currently, the incidence of PRES in the general pediatric population was found to be 0.4% reported by Thavamani et al. (8) in 2019. Our analysis showed that the risk of PRES in immunodeficient children increases significantly.

This study reported that acute CNS symptoms are likely common during ALL therapy in children. The CNS symptoms are frequent and occur most often during the first 2 months of treatment. CNS leukemic patients were at a higher risk for CNS diseases. In addition, in a total of 50 CNS patients, 29 patients (58%) had PRES. In the same cohort study, it was found that the cumulative incidence of T-cell leukemia was likely a higher risk than B-cell leukemia as reported by Banerjee et al. (57) in 2019. Moreover, about 29 to 57% of acute CNS symptoms have been reported during chemotherapy treatment (induction) in children (58–60). However, in two previous studies, the incidence of PRES in children with leukemia was 1.6–3.95% (59, 61). Additionally, the two new findings suggested that CNS involvement/leukemia was related to a significantly higher risk of PRES as compared with no CNS involvement/ leukemia. T-cell leukemia had a higher risk of PRES in pediatric hematologic disease after conducting chemotherapy (2, 20). The study on the risk factors of the incidence between PRES related to T-cell leukemia and CNS leukemia/involvement in our analysis showed that the risk of PRES in children with T-cell leukemia significantly increases. CNS leukemia/involvement has a significant role in promoting the occurrence of PRES in children.

### Strength and Limitations

Most of the six included studies, totaling 828 participants, are systematic reviews to investigate the factors associated with PRES in pediatric oncology after conducting chemotherapy and HSCT. To our knowledge, this is by far the most comprehensive research of the risk factors of pediatric treatment, including chemotherapy and HSCT, inducing PRES in the oncologic groups by using the meta-analysis.

However, it also has several limitations that have to be considered with caution when interpreting the result. Due to the lack of a full-text article, one of our six included studies in this review was selected from an editorial (27), and only eight factors, namely, sex (male), age group ≥10 years old (or 10.0–20.0 years), acute GVHD, hypertension, immunodeficiency, sickle cell disease, T-cell leukemia, CNS leukemia/involvement, and PRES outcomes, were evaluated; however, because of the lack of uniform standards or detailed information, some potential factors were excluded from this meta-analysis. Moreover, we have only conducted an inclusive search and screening using PubMed, Embase, and Web of Science databases. Furthermore, due to the heterogeneity among the studies included in this review, only observational studies were added, and the sample size of included studies was relatively insufficient, revealing the limited research in which the association between PRES and risk factors is examined. Despite the fact that our review was limited by the quality of studies reported from the pediatric oncologic diseases conducted with HSCT or chemotherapy, there may have been other relevant diseases (renal disorder, non-malignant disease, etc.) that were not eligible but included, especially (8). For a clear understanding on risk factors of PRES in pediatric oncologic diseases, we suggest that multicenter approaches in obtaining larger sample sizes may be necessary in analysis.

## Conclusions

In summary, this meta-analysis suggests that the risk in female children was significantly higher than in male children in the oncologic age groups of PRES. Children over the age of 10 years old in the oncologic age groups develop a significantly increased risk of PRES. Hypertension can promote the occurrence of PRES in children. Acute GVHD has a significant promotive effect on the occurrence of PRES. Children with SCD have a significantly increased risk of PRES. The risk of PRES in immunodeficient children increases significantly. The risk of PRES in children with T-cell leukemia rises considerably. CNS leukemia/involvement has a significant role in promoting the occurrence of PRES in children. This conclusion is based on a qualitative review of the six included studies that were confirmed by a meta-analysis. Based on the results above, it is expected that HSCT or chemotherapy for oncologic/hematologic diseases could be developed in the future to prevent PRES in children.

## Data Availability Statement

All datasets presented in this study are included in the article/ Supplementary Material.

## Author Contributions

MH, JT, MX, and CW: study design, idea, and writing. MH, PH, and MX: statistics and methods. JT, ZS, AA, and WW: producing data. All: formatting and editing the manuscript. All authors contributed to the article and approved the submitted version.

## Conflict of Interest

The authors declare that the research was conducted in the absence of any commercial or financial relationships that could be construed as a potential conflict of interest.
