# Supplementary figures and images for: Analysis of Risk Factors Associated With Poor Outcome in Posterior Reversible Encephalopathy Syndrome After Treatment in Children: Systematic Review and Meta-Analysis
Source: Front Neurol. 2020 Aug 26;11:938. doi: 10.3389/fneur.2020.00938 (PMC7479335; doi:10.3389/fneur.2020.00938)

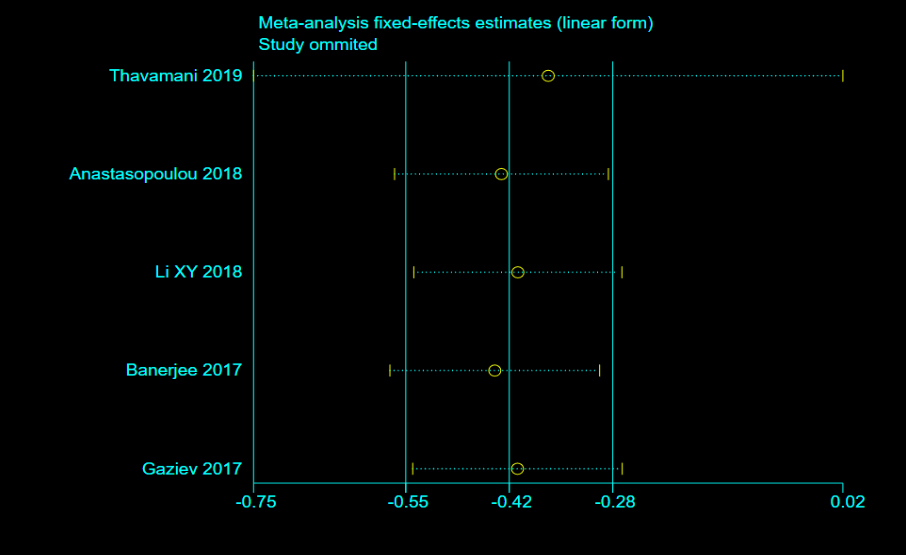

Supplement: Supplementary file 1 [file Image_1.JPEG]

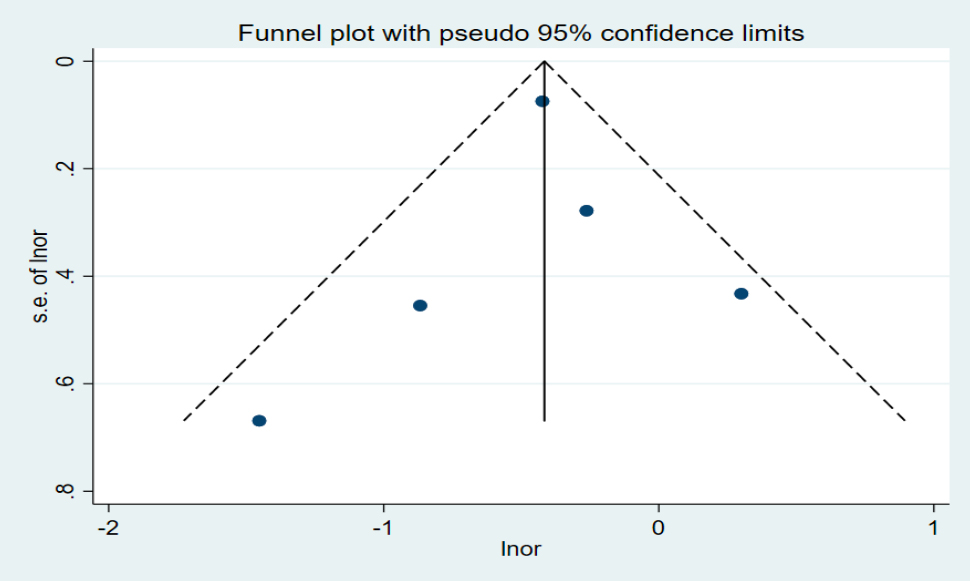

Supplement: Supplementary file 2 [file Image_2.JPEG]

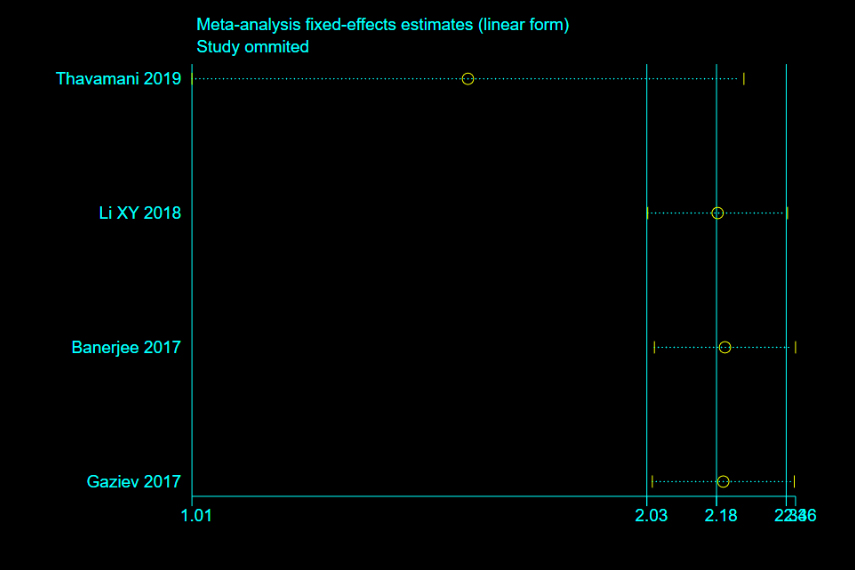

Supplement: Supplementary file 3 [file Image_3.JPEG]
